# Effects of Sound Interventions on the Mental Stress Response in Adults: Scoping Review

**DOI:** 10.2196/69120

**Published:** 2025-03-24

**Authors:** Marina Saskovets, Irina Saponkova, Zilu Liang

**Affiliations:** 1 Faculty of Engineering Kyoto University of Advanced Science Kyoto Japan; 2 Department of Psychology St Petersburg University St. Petersburg Russian Federation

**Keywords:** mental stress, anxiety, sound therapy, music therapy, psychoacoustics, expressive sounds, stress reduction, stress management, stress relief, stress markers, relaxation, personalized therapy, PRISMA

## Abstract

**Background:**

This scoping review examines the effects of various sound interventions, including music, natural sounds, and speech, on the stress response in adults.

**Objective:**

The review aims to identify key therapeutic factors, including sound type, individual listener characteristics, and environmental influences. It also synthesizes evidence on physiological responses to sound interventions and highlights current research gaps.

**Methods:**

We conducted a comprehensive search using databases such as PubMed, Web of Science, Scopus, and PsycINFO, focusing on studies from 1990 to 2024. Eligible studies included randomized controlled trials, clinical trials, and laboratory experiments that measured stress through physiological markers (eg, heart rate variability and cortisol) and self-reports. A total of 34 studies were included, and thematic analysis was conducted to identify recurring themes in the findings.

**Results:**

The findings suggest that music, especially classical and self-selected pieces, effectively reduces physiological stress markers, including cortisol levels, heart rate variability, and blood pressure. Nonmusical sounds, such as nature sounds and calming voices, also demonstrate potential for stress relief, although research in this area remains limited. While most sound interventions showed positive effects, some studies reported adverse effects, indicating that sound can both alleviate and induce stress. The outcomes were substantially affected by contextual factors such as personal preferences, delivery methods, cultural context, and emphasizing the importance of personalized interventions.

**Conclusions:**

Sound interventions offer promising, noninvasive methods for stress reduction. This review suggests that future research should address gaps in the study of nonmusical sound interventions and further investigate the neural mechanisms underlying stress responses to sound.

**International Registered Report Identifier (IRRID):**

RR2-10.2196/54030

## Introduction

### Background

Sound plays a fundamental role in human life, influencing communication, emotional regulation, and environmental awareness. Beyond its basic functions in speech and music, sound can modulate psychological and physiological states [1,2]. Auditory stimuli, including natural sounds, music, and human voices, have been shown to evoke a range of emotional responses and affect stress levels [3]. Recent research has explored the therapeutic potential of sound-based interventions, particularly in managing stress and promoting relaxation [4]. Much evidence documented a sound integrative impact on the psychoemotional and physiological outcomes, making it helpful for treating stress-related conditions such as pain syndromes or anxiety [5-7].

### Music and Music Therapy

Sound therapy techniques have gained prominence over the past decades, with a significant emphasis on music as a primary form of sound stimulation. One of the early definitions of music describes it as “humanly organized sound” [8], but there are several alternative perspectives debating this definition [9]. For example, Thaut [10] proposed that music is a complex, time-ordered, and rule-based sensory language. Similar to language, music constitutes a universal part of all human cultures, made up of individual sounds that are structured and layered [11]. The healing effects of music on the mind and body resonate with ancient cross-cultural beliefs [12]. Nowadays, music therapy incorporates various elements of music, such as melody, rhythm, tempo, dynamics, and pitch, along with activities such as songwriting, improvisation, and singing, to promote patients’ physical and mental well-being.

### Nonmusical Sound Therapies

Despite the widespread popularity of music, evidence indicates that other sound types, including natural noises, chanting, and speech, can also exert therapeutic effects. For example, listening to poetry has been shown to alleviate symptoms of anxiety and stress [13-15]. Moreover, in various therapeutic frameworks, the quality of communication is often considered a crucial factor. The effectiveness of treatment hinges not only on the specific technique or theoretical knowledge but also on the establishment of a trusting and intimate rapport between the client and the therapist. Ackerman and Hilsenroth [16] indicate that human-rated factors such as warmth, interest, and curiosity, contribute positively to the development of a strong therapeutic alliance. Therapists use different vocal styles depending on the session’s phase, aligning their voices to meet the emotional and therapeutic needs of the interaction [17]. However, distinguishing the impact of content from that of speech acoustics in poetry or therapeutic conversations presents a challenge. A comprehensive understanding of the factors that contribute to the therapeutic influence of sound remains to be elucidated.

### Mechanisms of Sound Therapy

The theoretical foundation of how sound interventions can yield therapeutic change and positive outcomes encompasses multiple levels, with the most prominent factor being the learned cognitive response shaped by cultural context [18].

#### Four-Level Model

Clements-Cortes and Bartel [18] proposed a 4-level model, suggesting that sound can trigger a response due to a set of learned associations that vary from person to person. A particular tone or sound can activate meaningful memories and evoke emotions associated with recalled events [18]. This phenomenon, known as music-evoked autobiographical memory recall, activates related brain networks, potentially inducing shifts in mood and emotional responses [9]. Other responses to sounds are closely linked to our inborn perceptive patterns. Music, characterized by organized vibrations and rhythms, reflects early learned responses. This forms the foundation of our reactions to music—fast music feels exciting, while slow music feels calming. Similarly, louder sounds are associated with strength and boldness, while quieter sounds seem weaker and softer [18].

These principles extend to nonmusical sounds: slower tempo-rhythmical structures, such as a gentle voice or soft breeze, can feel calming, while faster or louder sounds, such as a storm and thunder, can create higher arousal, excitement, or urgency [19,20]. In addition to inborn and early learned responses, situational emotional learning also shapes sound perception. Bliss-Moreau et al [21] demonstrated that voices can gain emotional meaning through past experiences, influencing how quickly participants respond to emotionally charged words based on their previous associations.

Levels 3 and 4 of the model by Clements-Cortes and Bartel [18] encompass responses to sound that are not the result of cognitive processing or learning but rather occur at a vibrational, rhythmic structure. Level 3 focuses on neural oscillatory coherence, where neurons fire in synchrony in response to rhythmic stimulation of the senses (auditory, visual, and tactile), which can lead to various beneficial effects. Level 4 suggests that music and sound can activate mechanisms at the cellular level, potentially influencing everything from neurons to bone and blood cells [18]. Thaut [10] indicates that the brain responds to sound rhythms through involvement, where the listener’s mood aligns with the emotional context conveyed in the sound. This occurs due to acoustic resonance, where the brain naturally synchronizes with the rhythm stimuli [10].

#### Role of Context and Common Therapeutic Factors

In practical applications, it is crucial to consider collateral factors that influence the perception of sound. The effectiveness of music as an art therapy method also depends on the surrounding environment and common factors applicable across creative therapies. These factors include being present in the moment, experiencing a predictable environment, feeling personally connected, developing social skills, finding meaning, feeling motivated, experiencing emotional release, and being actively engaged. Music therapy enhances therapeutic alliance and group processes through playful interactions, shared experiences, musical attunement, synchronicity, and dialogue. Musical engagement also modulates one’s sense of time and space, fostering a state of flow or providing distraction from stress-inducing thoughts [22]. Another framework for understanding how any therapy, including music therapy, facilitates positive changes is the mediator and moderator model [23,24]. This model considers external factors that affect the strength or direction of the relationship between treatment and the outcome. Moderators may include client or therapist characteristics (eg, gender, ethnicity, and experience), the format of the treatment (individual vs group and in-person vs online), or the treatment frequency (once vs twice a week) [25,26].

### Objective and Contribution of This Review

This scoping review aimed to analyze literature on sound interventions targeting stress response and stress-related conditions in adults in laboratory experiments, clinical trials, and randomized controlled trials (RCTs). We also aimed to identify sound interventions that have not been sufficiently studied and require further investigation.

We incorporated investigations focusing on responses of the hypothalamic-pituitary-adrenal axis and the autonomic nervous system as indicators of stress, supplemented by self-reported data and introspective surveys as markers of emotional stress. Our primary outcome of interest is the neural mechanisms underpinning the therapeutic influence of sound. In addition, we are interested in the comparison of delivery methods and sound sample choices for understanding the therapeutic factors of sound for stress reduction.

This study marks a conceptual shift in research of sound therapeutic effects by moving beyond music-focused frameworks to consider nonmusical acoustic interventions using human voices or environmental sounds. This broader perspective opens new possibilities for stress management strategies. By highlighting how diverse sounds can serve for therapeutic changes, this review encourages future studies to explore nonmusical sounds more rigorously and investigate how personal, cultural, or environmental contexts can interact with various sound types.

Moreover, this shift is expanding sound therapeutic potential to services such as classical therapeutic methods that use speech and conversation as primary tools. For instance, it is possible to consider the impact of vocal acoustics, such as tone or rhythm, within psychotherapy sessions to enhance emotional attunement and therapeutic alliance.

In addition, this concept can become a basis for the new technological development. It is possible to bring into the light, track, and analyze vocal elements in real time, allowing therapists to adapt their approach based on the client’s stress markers. Environmental sound tracking also holds potential for improving well-being, as ambient soundscapes could be monitored and adjusted to promote relaxation for everyday environments. This broader perspective encourages innovative approaches that personalize sound to individual needs.

## Methods

### Overview

This review follows the registered study protocol published in *JMIR Research Protocols* [27]. The protocol outlines the objective to comprehensively map empirical research on sound interventions for stress reduction. It adheres to Joanna Briggs Institute guidelines and follows the PRISMA-ScR (Preferred Reporting Items for Systematic Reviews and Meta-Analyses Extension for Scoping Reviews) checklist [28] (Multimedia Appendix 1), with minor adjustments, which will be detailed in the Protocol Adjustments subsection.

**Figure 1:**
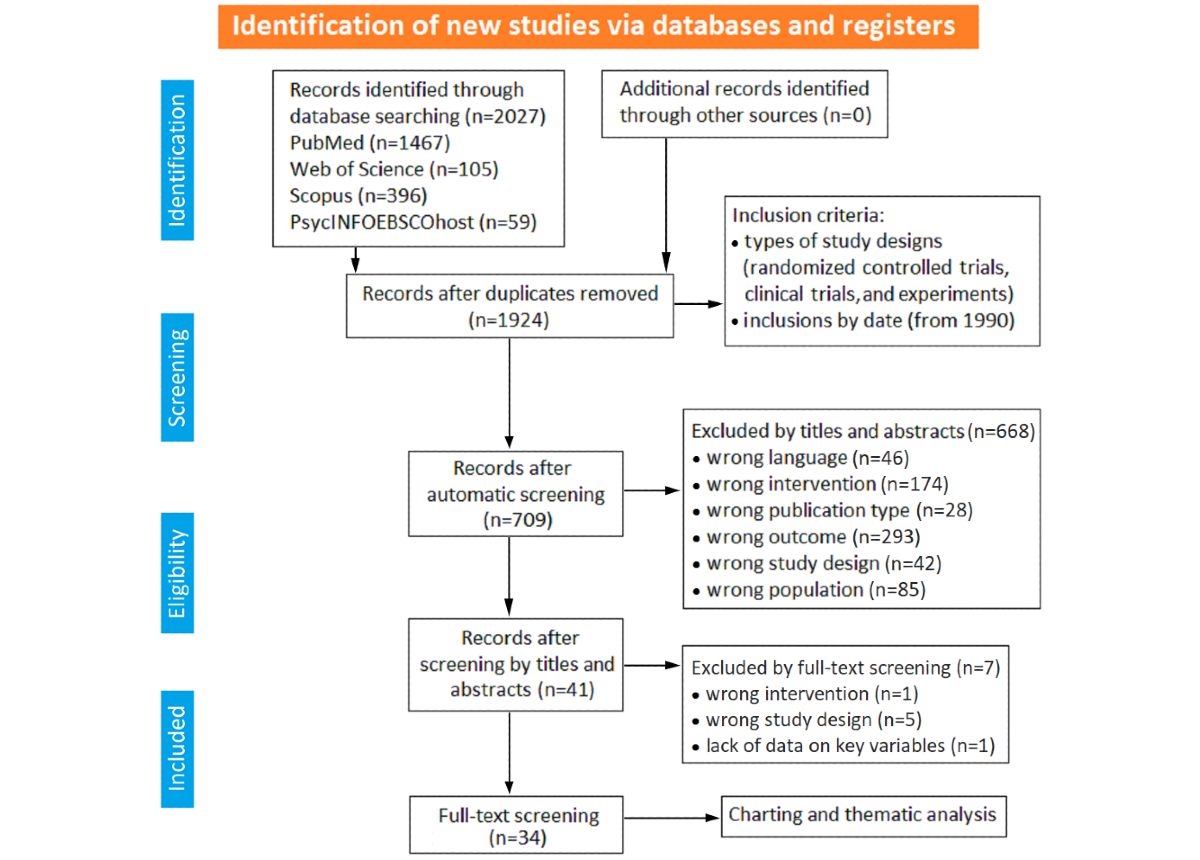
PRISMA-ScR (Preferred Reporting Items for Systematic Reviews and Meta-Analyses Extension for Scoping Reviews) flow diagram. It illustrates the study selection process, including the number of records identified, screened, excluded, and ultimately included in the final analysis [29].

The study protocol defines the eligibility criteria for study inclusion, focusing on adult participants, various sound interventions (eg, music, speech, and nature sounds), and stress-related outcomes. The PRISMA-ScR flow diagram (Figure 1) illustrates the study selection process. Guided by the protocol, this scoping review assesses the comparative effects of sound interventions, examines research directions, and identifies methodological challenges in existing literature.

### Research Questions

This scoping review was guided by the population, concept, context framework, recommended for constructing clear objectives and eligibility criteria in scoping reviews [29]. The population, concept, context framework in this study focuses on the population of interest (adults experiencing stress), the concept (therapeutic sound interventions), and the context (various settings in which sound interventions are applied for stress reduction). Through careful refinement, we formulated the following research questions to guide the entire review process:

1. What are the therapeutic factors of sound in the case of reducing stress response and stress-related conditions in human adults? For instance, it might be rhythm, sentiment, environmental context, personal preferences, or background.
2. What are the physiological responses associated with sound interventions in stressful conditions, as measured by biomedical technologies and devices?

### Search Strategy and Query String

This scoping review used a systematic search strategy across 4 key medical and interdisciplinary databases: PubMed, Web of Science, Scopus, and PsycINFO (or EBSCOhost). As the neurobiology of music and sound therapy emerged as a separate field in the 1990s, the search strategy covers the research from 1990 till the present day. The search aimed to capture studies addressing the effects of sound interventions, such as music therapy and guided relaxation, on stress reduction in adults. The complete query string formulated for this purpose was as follows**:** “(stress OR anxiety OR relax*) AND (“sound therapy” OR “music therapy” OR “guided relaxation” OR “guided meditation” OR hypno* OR ASMR OR MBSR) AND (prosody OR song OR poetry OR voice OR paralinguistics OR paralanguage) NOT (children OR infants OR animal OR teen).”

Using the operator “AND” we combined three key fields to comprehensively capture relevant literature (1) mental stress or relaxation indicators: terms such as “stress,” “anxiety,” and “relax*” were included to identify studies focused on both stress-related conditions and relaxation as therapeutic outcomes; (2) sound-based interventions: this field targeted interventions where sound is essential, encompassing “sound therapy,” “music therapy,” “guided relaxation,” “guided meditation,” “hypno*,” “ASMR,” (autonomous sensory meridian response) and “MBSR” (mindfulness-based stress reduction); and (3) voice and paralinguistic elements: we included terms, such as “prosody,” “song,” “poetry,” “voice,” “paralinguistics,” and “paralanguage” to capture studies examining the acoustic characteristics of speech and natural language as therapeutic components. The exclusion criteria were applied using the NOT operator to filter out studies on children, infants, animals, and adolescents, ensuring a focus on adult populations.

This structured approach aimed to capture a comprehensive range of studies, not only focusing explicitly on sound-based therapies but also those involving therapeutic aspects of speech and vocal elements. By broadening the scope in this way, we aimed to explore potential blind spots in sound therapy research, particularly in the therapeutic roles of natural speech and paralinguistic features alongside traditional music-based interventions.

### Study Selection

The process of how citations and full-text reports were reviewed, included, and excluded is shown in the PRISMA-ScR flow diagram (Figure 1). The first author, a clinical psychologist, led all aspects of the review, including literature search, extraction, screening, and data analysis. The second author, a psychology researcher, assisted in the literature search and extraction and performed screening independently. Disagreements were resolved through discussion and consensus with other authors of the research consortium.

A 2-step process was followed for study selection. First, we screened citation titles, abstracts, and keywords, classifying each citation as “include,” “exclude,” “unclear,” or “duplicate.” In the second step, full-text reports for citations marked “include” and “unclear” were reviewed to make a final decision on inclusion or exclusion. Reference management and screening were conducted in Rayyan (Rayyan Systems Inc), a web-based app for systematic review management. The inclusion and exclusion criteria are presented in Tables 1 and 2. The inclusion criteria were used to select studies for the review, specifying population, intervention type, outcome measures, study design, and publication type. The exclusion criteria outline conditions under which studies were excluded, including participant characteristics, health conditions, intervention types, and methodological limitations. These criteria ensure a focused review on adult populations using diverse passive sound interventions for stress relief, with an emphasis on studies providing measurable physiological data.

**Table 1:** Inclusion criteria used to select studies for the review, specifying population, intervention type, outcome measures, study design, and publication type.

Inclusion criteria	General description and examples
Population	Human adults exposed to stress-related conditions, including both clinical and healthy populations. Examples include the following: Anxiety, reactive depression, crises and emergency cases, grief, loss, basic needs deprivation, burnout, occupational hazards Experimental stress
Intervention type	All types of passive sound interventions. Examples include the following: Listening to instrumental music, songs, poetry, human voices, and nature sounds Sound-based therapies, including music therapy, acoustic stimulation during relaxation, meditation, and induction of hypnosis
Outcome measurements	Studies that assess stress relief effects through physiological measures (eg, heart rate variability, salivary cortisol, electrodermal activity, neuroimaging markers) Studies that combine both physiological measurements and self-reported stress levels (eg, Perceived Stress Scale, State Anxiety Inventory, Beck Anxiety Inventory)
Study design	Randomized controlled trials Clinical trials Laboratory experiments
Publication type	Peer-reviewed original research articles published in English

**Table 2:** Exclusion criteria, including participant characteristics, health conditions, intervention types, and methodological limitations.

Exclusion criteria	General description and examples
Population	Participants younger than 18 years Animals Individuals with hearing disabilities
Health conditions	Conditions for which stress is not the original cause. Examples include the following: Pain syndromes Psychotic episodes Chronic neurological disorders, such as Alzheimer or Parkinson disease Age-related cognitive changes Autism spectrum disorders Tinnitus, hyperacusis
Outcome measurements	Studies relying solely on questionnaires or self-reports without physiological measurement data
Intervention type	Active sound interventions, including improvisation, music composition, drumming, singing, poetry writing, or chanting Mixed interventions that combine sound with unrelated therapeutic interventions, such as aromatherapy or massage Mono-interventions: studies in which sound (usually an unclearly defined piece of music) represented the only intervention, and the absence of sound represented the comparator

### Data Extraction

After initial screening according to the chosen criteria, 2 reviewers independently evaluated the full-text articles. Key information included the following items: (1) authors and year of publication indicating the first author and publication year; (2) country of origin specifying the country where the study was conducted; (3) concept specifying the main idea and hypothesis of the study; (4) population specifying the sample size and population type (eg, nurses, students, and patients with specific diagnoses); (5) study type, for instance, RCTs, clinical trials, or laboratory experiments; (6) intervention type (groups) indicating the type of sound stimulation as described in the article (eg, music, voice, and nature sounds). In most cases, the separation of participants into groups is based on the intervention. This is where we get information about both the types of sound interventions and the participant grouping; (7) outcomes and measurements specifying the target condition or issue that the sound stimulation aims to impact, and how it was measured, including both physiological measures and self-reports of subjective experience (eg, measurements of stress hormones, heart rate variability (HRV), anxiety scale, and emotional state questionnaires); and (8) key findings related to the scoping review questions—indicating the primary results, such as clinical outcomes, body-mind responses, effectiveness, among others.

### Thematic Analysis Method

The thematic analysis for this scoping review followed the reflexive thematic analysis framework by Braun and Clarke [30], using an inductive approach to identify key therapeutic factors of sound interventions on stress reduction. The analysis began with a familiarization phase, in which the first 2 authors thoroughly reviewed and familiarized themselves with the data extracted from each included study. This initial stage focused on understanding the study concept and intervention types.

Following familiarization, an open coding process was conducted across the entire dataset, centering on essential elements, such as sound characteristics, and individual outcomes. This coding identified basic elements such as music styles, sentiment, basic physical elements (rhythm and frequency), environmental context, and personal context as core factors within the data. Related codes were then grouped into preliminary categories, organizing the themes based on factors influencing the effectiveness of sound interventions. Three broad categories emerged from this process: (1) *sound per se*, capturing sound characteristics independent of the listener; (2) *personal factors*, which focused on listener-dependent characteristics, including musical education, cultural background, personal preferences, and stress history; and (3) *environmental factors*, covering aspects of sound delivery, such as live versus recorded sound, use of headphones or broadcast sound, and interactions with medical staff or researchers during the session.

Within the category of *sound per se*, which proved to be the most widely researched, 9 subcategories were developed to reflect specific sound types and qualities. The first 3 of them (1-3) related to music, including different styles, sentiment (such as cheerful vs sad music), and elementary characteristics (such as tempo-rhythm), the next 3 (4-6) outlined nonmusical sounds (eg, white noise, nature sounds, and human voices), and 3 others (7-9) related to the comparison of musical and nonmusical sounds. Initially, the category “environmental context” included subcategories such as “live versus recorded sound” and “presence of supportive personnel,” while “personal preferences” encompassed elements such as “music genre” and “cultural background.” At the same time, due to the modest number of articles assigned to these themes, we decided not to overload the chart and did not include these subcategories in the final edition. In addition, it was important for us to add 3 extra categories for outcomes that were out of the main pattern or for negative outcomes. These were mixed samples (“mix”), relaxing effects of the absence of sound (“0”), and negative, stressful sound effects (“–”).

During the thematic refinement stage, categories and subcategories were iteratively adjusted through regular team discussions and codebook updates, enhancing internal coherence and consistency across the themes. As the thematic map took shape, each category was further refined and defined to ensure clarity and distinctiveness. This thematic framework not only provided a structure for categorizing therapeutic factors and mechanisms of sound interventions but also revealed patterns in the literature and highlighted gaps for future research. As shown in the Results section, some categories have little research, suggesting areas for future studies. We found that in most of the studies reviewed, the hypotheses and groupings were based on the type of sound intervention. This means that the groups typically reflect the type of sound being tested. In some cases, there were >2 types of sounds. If the categorization was unclear or debated, we referred to the study’s concept and research question to identify the primary focus of authors. For instance, if a study had 3 groups, “cheerful music,” “sad music,” and “white noise,” we would place it in the “musical sentiment” subcategory as the study’s focus is on musical emotion rather than comparing music to nonmusical sounds.

### Protocol Adjustments

This section outlines the adjustments made during the review process compared to the study protocol [27]. While the overall methodology remained consistent, some procedural differences emerged during implementation. One notable change was the exclusion of gray literature sources, such as Google Scholar, ClinicalTrials.gov, and nonindexed conference proceedings. The final review focused exclusively on peer-reviewed studies to maintain methodological rigor. Many gray literature sources lacked transparency in their methods, making it difficult to assess their reliability. In addition, most did not report detailed physiological stress markers, such as cortisol levels, HRV, or neuroimaging data, which were essential for inclusion in this review. Instead, they often emphasized subjective well-being and relaxation effects, which, while relevant, did not align with the primary focus of this study*.* Given these challenges, the review prioritized published studies that had undergone formal peer review, ensuring that all findings were based on reliable findings. Despite these adjustments, the core thematic analysis approach, guided by the framework by Braun and Clarke [30], remained unchanged. These refinements ensure that the findings accurately reflect the available evidence while maintaining alignment with the original research objectives.

## Results

### Overview

The systematic database search obtained 2027 records. After removing duplicates, 1924 records remained for further review. Screening of titles and abstracts led to the exclusion of records that did not meet the chosen criteria, such as unsuitable languages, outcomes, or interventions. Full-text review of the remaining 41 records revealed that 7 did not meet the inclusion criteria due to various reasons, including wrong intervention (n=1, 2%), wrong study design (n=5, 12%) and lack of data on key variables (n=1, 2%). Finally, 34 studies were considered eligible for inclusion in the analysis. These studies were RCTs (22/34, 65%), experiments (9/34, 26%), and pilot studies (3/34, 9%). The search process is outlined in the PRISMA-ScR flowchart (Figure 1). A summary of the included studies, detailing their country of origin, research concept, participant population, and study design is outlined in Table 3.

**Table 3:** An overview of the included studies.

Study and year	Country	Concept	Population	Study type
Umemura and Honda [31], 1998	Japan	Classical music may promote relaxation by influencing heart rate variability compared to rock music or noise.	6 university students, aged between 21 and 26 years	Experimental study
Chafin et al [32], 2004	United States	Listening to classical music may improve blood pressure recovery after a stressful task compared to silence or other music styles.	75 healthy participants	Experimental study
Labbé et al [33], 2007	United States	Listening to classical or self-selected relaxing music reduces anxiety, anger, and physiological arousal after stress compared to heavy metal music or silence.	56 college students	RCT^a^
Uğraş et al [34], 2018	Turkey	All types of music can reduce preoperative anxiety, with classical Turkish music being the most effective.	180 patients undergoing surgery	RCT
Gulnahar and Kupeli [35], 2020	Turkey	Listening to music reduces anxiety during dental implant surgery, with Turkish music and classical music potentially being most effective.	80 dental implant surgery patients, aged between 40 and 70 years	Prospective, observational RCT
Paszkiel et al [36], 2020	Poland	Relaxing music and autonomous sensory meridian response music may reduce stress levels faster than silence or rap music. Rap music may even worsen stress compared to silence.	9 healthy female participants, aged 22years	Pilot study
Hirokawa and Ohira [37], 2003	Japan	High-uplifting and low-uplifting music may have different effects on immune function, neuroendocrine responses, and emotional states after a stressful task.	18 Japanese college students	Experimental study
Sokhadze [38], 2007	United States	Both pleasant and sad music may improve physiological recovery after stress (viewing stressful images) compared to white noise. White noise may not enhance recovery.	29 healthy participants	Experimental study
Suda et al [39], 2008	Japan	Major mode music reduces stress more than minor mode music.	10 graduate students	Experimental study
Wiwatwongwana et al [40], 2016	Thailand	Music may reduce anxiety in patients undergoing cataract surgery. Binaural beat audio may offer additional benefits over music alone.	141 patients undergoing cataract surgery under local anesthesia	Prospective, double-blind RCT
Opartpunyasarn et al [41], 2022	Thailand	Binaural beat audio may reduce anxiety in patients undergoing fiber-optic bronchoscopy compared to plain music or no music.	112 patients undergoing fiber-optic bronchoscopy	Prospective RCT
Lee-Harris et al [42], 2018	United Kingdom	Meditative binaural music may be effective for relaxation, with effects potentially differing by age.	30 (15 and 15) participants, aged between 18and 25 years and 50 and 80 years	RCT
Gantt et al [43], 2017	United States	Binaural beat technology (theta waves) embedded in music may be more effective than music alone in reducing cardiovascular stress response in military personnel with postdeployment stress.	74 military service members with postdeployment stress	Double-blind RCT
Calamassi et al [44], 2022	Italy	Listening to music at 432 Hz and 440 Hz may reduce anxiety and stress biomarkers compared to a no-music control group.	54 emergency nurses	Double-blind RCT
Sharma et al [45], 2021	India	Indian classical music with incremental tempo and octave variations promotes better anxiety reduction.	21 male undergraduate medical students	Crossover RCT
Singh et al [46], 2009	India	Both music and progressive muscle relaxation are effective in reducing anxiety and dyspnea in hospitalized patients with COPD^b^. Music may be more effective than PMR^c^ for reducing anxiety.	72 hospitalized patients with COPD with recent exacerbation	RCT
Tang et al [47], 2009	United States	Audio relaxation programs may be effective for short-term blood pressure reduction in older adults, potentially more effective than listening to Mozart.	41 older adults	RCT
Lin et al [48], 2011	Taiwan	Music therapy and verbal relaxation are effective in reducing anxiety induced by chemotherapy.	98 patients with cancer under chemotherapy	RCT
Lee et al [49], 2012	Germany	Both monochord sounds and progressive muscle relaxation can reduce anxiety and improve relaxation during chemotherapy.	40 patients with gynecologic cancer under chemotherapy	RCT
Warth et al [50], 2016	Germany	Live music therapy may be more effective than a prerecorded mindfulness exercise in improving cardiovascular health for patients who are terminally ill through its influence on the autonomic nervous system.	84 patients in palliative care	RCT
Koehler et al [51], 2022	Germany	Music therapy may be more effective than mindfulness in reducing subjective distress in patients in palliative care. Both interventions may reduce stress biomarkers.	104 patients in palliative care	RCT
Radstaak et al [52], 2014	Netherlands	Listening to self-chosen relaxing or happy music after stress may improve mood but delay systolic blood pressure recovery.	123 healthy participants	Experimental study
Leardi et al [53], 2007	Italy	Music therapy, especially patient-selected music, may reduce stress response during day surgery compared to no music.	60 patients undergoing day surgery	RCT
Miller et al [54], 2010	United States	Music may influence endothelial function, potentially improving vascular health.	10 healthy participants, average age 35.6 years	Crossover RCT with counterbalancing
Imbriglio et al [55], 2020	Canada	Guided music listening with relaxing music or a participant’s favorite music may decrease muscle activity and bruxism episodes in chronic myalgia, while stressful music may increase them.	14 women with chronic myalgia and 15 women who are pain free	Experimental study
Gelatti et al [56], 2020	Italy	Live harp music may be more effective than recorded harp music in reducing preoperative stress, fear, heart rate, and blood pressure.	46 patients undergoing day surgery	Pilot, quasi-experimental study
Bro et al [57], 2019	Denmark	Live music may reduce anxiety during chemotherapy compared to prerecorded music or standard care.	143 newly diagnosed patients with lymphoma	Multicenter RCT
Lee et al [58], 2011	Taiwan	Both headphones and broadcast music can effectively reduce preoperative anxiety in adult patients undergoing surgery.	167 patients undergoing surgery without premedication	RCT
Kumari et al [59], 2023	India	Broadcast and headphone music playing may vary the anxiety-relieving effect for patients awaiting surgery.	150 healthy participants	Experimental study
Lai et al [60], 2012	Taiwan	Music intervention with a nurse present is more effective than recorded music alone for improving psychophysiological health in caregivers for patients with cancer.	34 female caregivers for patients with cancer	Crossover RCT
Janelli et al [61], 2004	United States	Listening to preferred music may reduce negative behaviors in patients who are physically restrained.	30 patients who are physically restrained, aged between 65 and 93 years	Pilot study
Kang et al [62], 2008	South Korea	Blocking noise, but not music, reduces bispectral index scores during propofol sedation in noisy operating rooms.	63 patients undergoing total knee replacement surgery, aged between 55 and 75 years	Prospective, single-blind RCT
Tsivian et al [63], 2012	United States	Music with headphones may reduce pain perception and anxiety during prostate biopsy compared to control or headphones alone.	88 men undergoing transrectal ultrasound prostate biopsy	Prospective RCT
Gingras et al [64], 2014	Austria	Repetitive drumming with shamanic instructions may affect subjective experiences and cortisol levels compared to instrumental meditation music.	39 participants inexperienced in shamanic journeying	Experimental study

^a^RCT: randomized controlled trial.

^b^COPD: chronic obstructive pulmonary disease.

^c^PMR: progressive muscle relaxation.

### Thematic Analysis Results

**Figure 2:**
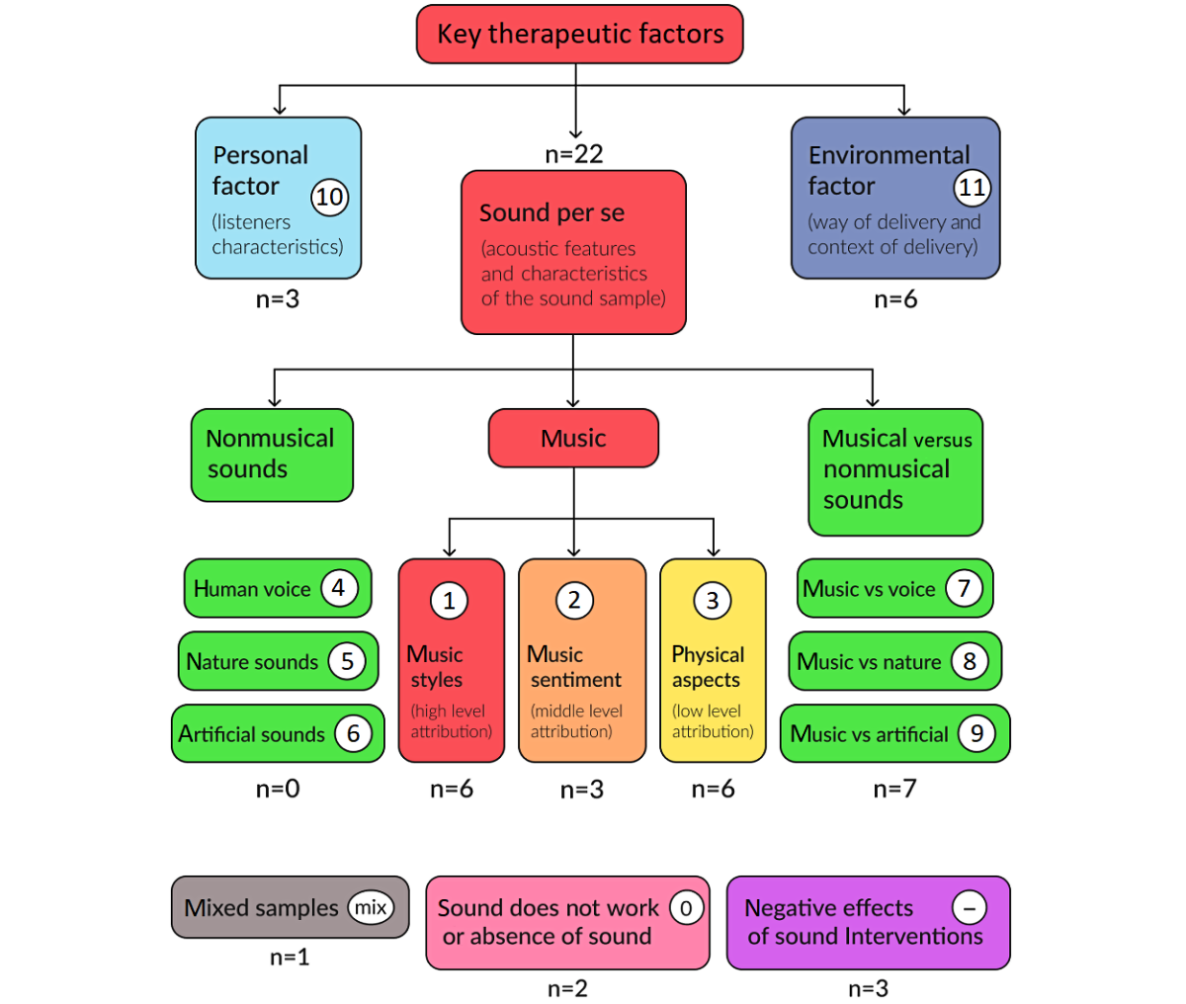
A summary of the thematic analysis, categorizing sound interventions into key therapeutic factors, including sound characteristics, personal factors, and environmental influences on stress reduction. The numbers in white circles represent classification labels assigned to categories based on thematic analysis (see the Thematic Analysis Method under Methods for details).

Thematic analysis of the included studies revealed several key factors contributing to the effectiveness of sound interventions. These factors can be categorized into 3 main categories: *sound per se*, *personal factors*, and *environmental factors* (Figure 2).

The majority of the identified articles focused on the characteristics of sound per se (22/34, 65%). For example, studies investigating music interventions often explored different music styles (6/34, 18%), music sentiment (3/34, 9%), and physical aspects such as frequency or tempo (6/34, 18%).

Regarding nonmusical sounds within our predefined inclusion criteria, we found publications comparing musical interventions with a variety of voice-assisted interventions (7/34, 21%). However, no studies were found that focused narrowly on nonmusical sounds for stress relief in adults or compared different sound characteristics beyond the concept of musical intervention.

Within the personal factors category, listener characteristics (3/34, 9%) were identified as influencing intervention outcomes. Environmental factors, such as the way of delivery and context of delivery, were also considered (6/34, 18%).

In addition, the analysis identified a small number of studies (1/34, 3%) that explored mixed samples of sounds and a few that shifted their focus from sound to silence (2/34, 6%) or reported negative effects of sound intervention (3/34, 9%).

Information on key findings for comparing the effectiveness of different sound intervention factors is summarized in Table 4.

**Table 4:** The effects of sound interventions.

Study and year	Interventions (groups)	Category	Outcomes (measurements)	Key findings
Umemura and Honda [31], 1998	Classical music Rock music Noise Rest	1	HRV^a^ (MWSA^b^ and RSA^c^ components) and subjective comfort levels (psychological evaluation)	Classical music reduced variability in HR^d^ components (MWSA and RSA) compared to rest, suggesting a potential relaxation effect. Rock music and noise increased MWSA and decreased RSA, potentially indicating a stress response. Subjectively, classical music was associated with comfort, while rock music and noise were associated with discomfort. Changes in MWSA correlated with comfort levels, suggesting a link between HRV and perceived comfort.
Chafin [32], 2004	Classical music Jazz music Pop music No sound (control)	1	SBP^e^ measured before and after the stressful task.	Listening to classical music resulted in significantly lower posttask SBP compared to silence. Other music styles did not show significant benefits for blood pressure recovery.
Labbé et al [33], 2007	Classical music Self-selected relaxing music Heavy metal music No sound (control)	1	Emotional state (anxiety and anger) and physiological arousal (before and after intervention)	Listening to self-selected or classical music significantly reduced negative emotions and physiological arousal compared to heavy metal music or silence.
Uğraş et al [34], 2018	Turkish classic music Western music Natural sounds No sound (control)	1	STAI-S^f^) for anxiety, SBP, DBP^g^, HR, and cortisol levels measured before and after music intervention	All music types significantly reduced anxiety (STAI-S), SBP, and cortisol levels compared to before the intervention. Natural sounds reduced DBP, and classical Turkish music showed the most significant reductions in DBP and HR. All music was effective in reducing preoperative anxiety, with classical Turkish music being the most beneficial.
Gulnahar and Kupeli [35], 2020	Turkish classic music Classical music (Vivaldi) Slow rock music No sound (control)	1	Pre- and postintervention anxiety using the Corah Dental Anxiety Survey, blood pressure, HR, and oxygen saturation	All music groups showed reduced anxiety compared to controls. Turkish music and classical music were significantly more effective in anxiety reduction compared to slow rock music.
Paszkiel et al [36], 2020	Rap music Relaxing music ASMR^h^ music No sound (control)	1	EEG^i^–alpha wave activity, blood pressure, HR, and subjective stress perception questionnaires	Relaxing music and ASMR reduced stress levels faster than silence. Rap music increased stress levels compared to silence.
Hirokawa and Ohira [37], 2003	High-uplifting music Low-uplifting music No sound (control)	2	S-IgA^j^ level, active NK^k^ cell level, T lymphocyte subsets (CD4^+^, CD8^+^, and CD16^+^), dopamine, norepinephrine, and epinephrine levels (before and after intervention), and emotional state questionnaires (before and after intervention)	Results were inconclusive, but trends suggested (1) low-uplifting music may increase feelings of well-being; (2) high-uplifting music may increase norepinephrine and liveliness and decrease depression; and (3) silence may decrease active NK cells. Music classification is important for understanding music’s influence on these responses.
Sokhadze [38], 2007	Subjectively pleasant music Sad music White noise No sound (control)	2	EEG—frontal and temporal activity, skin conductance, HR, HRV, facial capillary blood flow, respiration rate (measured before and after stressful images and during recovery interventions)	Both pleasant and sad music improved recovery on most measures compared to white noise (HR, respiration rate, and blood flow). White noise did not enhance recovery. Both music types positively influenced cardiovascular and respiratory activity during recovery. The “undoing hypothesis” (positive emotions aid recovery from negative emotions) was partially supported.
Suda et al [39], 2008	Major music Minor music No sound (control)	2	Salivary cortisol levels (endocrine stress marker) and optical topography (brain activity)	Major mode music resulted in lower salivary cortisol levels (stress marker) compared to minor mode music. This suggests music can induce emotional responses such as happiness, potentially linked to stress reduction.
Wiwatwongwana et al [40], 2016	Binaural beat music Plain music No sound (control)	3	STAI score (before and after intervention), SBP, and HR (measured at admission, surgery start, and 20 min after surgery).	Both music with and without binaural beats reduced anxiety (STAI score) and SBP compared to the control group. Binaural beat music showed an additional decrease in HR compared to plain music or the control group. Binaural beat audio embedded in music may have some advantages over music alone for reducing anxiety during cataract surgery.
Opartpunyasarn et al [41], 2022	Binaural beat music Plain music No sound (control)	3	STAI score (before and after bronchoscopy), blood pressure, HR, and sedative use	Patients in the binaural beat group showed a significantly greater reduction in anxiety (STAI score) compared to the plain music and no music groups. They also experienced a decrease in blood pressure but an increase in HR. Binaural beat audio may be effective for reducing anxiety before bronchoscopy.
Lee-Harris et al [42], 2018	Meditative music with binaural beats Meditative music without binaural beats Low-arousal classical music High-arousal classical music	3	Self-reported emotional state (arousal and positivity) and physiological arousal (measured but not specified in the abstract).	The effect of listening to MBM^l^ was comparable to listening to calm classical music. Younger adults showed a stronger decrease in self-reported arousal with MBM with binaural beats compared to low-arousal classical music. Older adults showed a preference for low-arousal classical music for feeling comforted, followed by MBM. These results suggest age may influence how music affects relaxation.
Gantt et al [43], 2017	Music with embedded theta binaural beats Music alone	3	HRV was measured before and after the intervention to assess changes in sympathetic and parasympathetic nervous system activity. Daily self-reported stress levels were recorded in diaries.	Music with binaural beats led to a decrease in low-frequency HRV (indicating reduced sympathetic response) and an increase in high-frequency HRV (indicating increased parasympathetic response) compared to music alone. The binaural beats group also reported lower daily stress levels compared to the music-only group. Overall, music with theta binaural beats showed promise in reducing physiological and psychological stress markers in military personnel with postdeployment stress.
Calamassi et al [44], 2022	Listen to 440 Hz music during break Listen to 432 Hz music during break Usual break activities (control)	3	STAI-X1, HR, respiratory rate, SBP and DBP, and pain and productivity (Likert scale)	All groups showed a reduction in anxiety after the break. Listening to 432 Hz music showed the greatest reduction in anxiety and additionally reduced respiratory rate and SBP. Music at 440 Hz reduced anxiety but showed no significant physiological benefits in this study.
Sharma et al [45], 2021	VM^m^ with incremental tempo or octave changes SM^n^ without variations No sound (control)	3	Anxiety (Beck Anxiety Inventory and STAI), EEG for brainwave activity, ECG^o^ for HRV	Significant anxiety reduction only in the VM group. VM showed decreased low-frequency brainwaves and midline power (reduced default mode network activity) compared to silence. VM also showed more balanced brain activity compared to SM. HRV remained stable during music interventions. The authors propose VM induces a “controlled mind wandering” state leading to anxiety reduction.
Singh et al [46], 2009	Self-selected music Audio instructions (PM^p^)	7	STAI (Spielberger inventories), dyspnea, SBP and DBP, pulse rate, and respiratory rate (before and after intervention)	Both music and PMR significantly reduced anxiety, dyspnea, SBP, pulse rate, and respiratory rate. However, the music group showed greater reductions in anxiety compared to the PMR group. Music and PMR are effective for reducing anxiety and physiological measures in hospitalized patients with COPD^q^, with music potentially being more effective for anxiety reduction.
Tang et al [47], 2009	12-minute audio relaxation program 12-minute Mozart andante	7	Blood pressure (systolic and diastolic) was measured at each intervention session and at 1-month and 3-month follow-up	Both groups showed significant reductions in blood pressure after the intervention. The audio relaxation group showed a greater reduction in SBP than the Mozart group. Blood pressure reductions were not sustained at 1- and 3-month follow-up. Audio relaxation may be more effective than music for short-term blood pressure reduction in older adults.
Lin et al [48], 2011	1 hour session of music therapy 30 minutes of guided verbal relaxation Usual care	7	State anxiety (Spielberger STAI), emotional distress (Emotional Visual Analog Scale), and physiological responses (skin temperature, HR, and consciousness level) before and after chemotherapy	Music therapy was more effective than verbal relaxation or usual care in reducing postchemotherapy anxiety and increasing skin temperature. Patients with high initial anxiety benefited more from music therapy than those with normal anxiety. Both interventions showed some effectiveness within 30 minutes.
Lee et al [49], 2012	monochord sounds PMR	7	Spielberger State Anxiety Inventory for Anxiety, questionnaires on physical and psychological states, EEG data before and after intervention	Both the monochord sounds and PMR groups showed significant improvements in anxiety, physical, and psychological state. EEG data showed increased posterior theta and decreased midfrontal beta-2 activity in both groups, indicating relaxation. The monochord sounds group also showed a decrease in alpha band activity compared to PMR. Both interventions were effective for reducing anxiety and improving relaxation, with potentially different underlying neural mechanisms.
Warth et al [50], 2016	Live music therapy Prerecorded mindfulness exercise	7	Vagally mediated HRV and blood volume pulse amplitude were measured over time to assess autonomic nervous system response	Both groups showed improvements over time, but music therapy led to significantly greater reductions in vascular sympathetic tone (BVP-A^r^). This suggests music therapy may be more effective in managing pain and stress symptoms in palliative care. Baseline pain levels influenced patient response, highlighting its importance in treatment planning. Music therapy’s impact may be related to the therapist-patient relationship in addition to the music itself.
Koehler et al [51], 2022	3 sessions of music therapy 3 sessions of mindfulness training	7	Subjective distress rating scale (before and after intervention), salivary cortisol and alpha-amylase levels (before and after intervention), and HR and HRV	Music therapy showed a greater reduction in subjective distress compared to mindfulness. Both interventions led to reductions in cortisol and HR, but no significant differences between groups were found for these or other stress biomarkers.
Radstaak et al [52], 2014	Self-chosen relaxing music Self-chosen happy music Audiobook Silence (control)	7	Mood questionnaires, subjective arousal and rumination questionnaires, and SBP and DBP, HR.	Listening to self-chosen relaxing or happy music improved mood compared to control conditions. No significant effects on subjective arousal or rumination. Both relaxing and happy music groups showed delayed SBP recovery compared to control conditions.
Leardi et al [53], 2007	New age music Choice of music from 4 styles Control group (standard operating room sounds)	10	Plasma cortisol levels and subpopulations of lymphocytes (NK cells) were measured before, during, and after surgery	Both music groups showed decreased cortisol (stress hormone) levels during surgery compared to the control group. Patients who chose their music (group 2) had significantly lower postoperative cortisol levels than those who listened to new age music (group 1). NK cell levels (immune function) decreased during surgery in the music groups and increased in the control group. Patients listening to new age music (group 1) had lower levels of NK cells during surgery compared to the control group.
Miller et al [54], 2010	Listening to self-selected joyful or anxiety- provoking music for 30 minutes Watching video to induce laughter and listening to audio for relaxation	10	Endothelial function was measured by brachial artery flow-mediated dilation before and after each intervention	Joyful music increased flow-mediated dilatation (blood-vessel dilation), while anxiety-provoking music decreased flow-mediated dilatation. The increase in joyful music was comparable to improvements seen with exercise or medication.
Imbriglio et al [55], 2020	Relaxing music Favorite music Stressful music Pink noise	10	Electromyographic activity in the right masseter muscle to measure muscle effort: posture and spontaneous awake bruxism episodes and duration and frequency of awake bruxism episodes	Relaxing music and favorite music decreased muscle effort during awake bruxism in chronic temporomandibular disorders by 26% and 44%, respectively. Stressful music increased muscle effort. Guided music listening with selected music may be a promising noninvasive treatment for chronic temporomandibular disorders.
Gelatti et al [56], 2020	Live harp music intervention Recorded harp music control	11	Self-reported fear and stress levels (before and after intervention) and blood pressure (before and after intervention), (3) HR (before and after intervention)	Both live and recorded harp music significantly reduced fear. Live harp music was more effective in reducing HR and DBP compared to recorded music.
Bro et al [57], 2019	30 minutes of patient-preferred live music 30 minutes of patient-preferred prerecorded music Standard care	11	Anxiety was measured by the Spielberger State Anxiety Inventory, blood pressure, pulse rate, nausea, vomiting, serum catecholamine levels, and health-related quality of life. The Musical Ability Test was used to assess if musical ability influenced anxiety.	Live music showed a borderline statistically significant reduction in anxiety compared to standard care. Prerecorded music had no significant effect. No significant changes were observed in secondary outcomes. Musical ability did not influence the effect of music.
Lee et al [58], 2011	Headphone music therapy Broadcast music therapy Control (no music)	11	Visual Analog Scale for anxiety (before and after intervention) and HRV (before and after intervention)	Both headphones and broadcast music significantly reduced visual analog scale scores (anxiety) compared to the control group. No significant difference was found between headphones and broadcast music for reducing anxiety. Broadcast music offers an alternative to headphones for reducing preoperative anxiety while considering infection control.
Kumari et al [59], 2023	Headphone group Broadcast group Control	11	Preoperative anxiety by visual analog scale and HRV	The average HRs of the broadcast group, headphone group, and control group were not significantly different. The mean anxiety score of the control group was significantly higher than that of the headphone group and the broadcast group. Time-domain HRV was not significantly different. There was a significant difference in high-frequency HRV among the 3 groups. LF in the broadcast group was significantly lower than the control group, indicating there was less tension of the sympathetic nervous system in the broadcast group. Both headphones and broadcast music are effective for reducing the preoperative patient’s anxiety in the waiting room. Thus, when headphones are not available or not appropriate, speakers can be an effective substitute.
Lai et al [60], 2012	Music with nursing presence Recorded music	11	Blood volume pulse amplitude and HRV, depression, anxiety, and sleep quality were measured before and after intervention, and participant evaluation of music	Both interventions improved anxiety, depression, and blood volume pulse amplitude. Music intervention with a nurse led to greater reductions in anxiety and improved ease of falling asleep compared to recorded music. Participants preferred music with a nursing present and found it to be more harmonious and friendly. Both interventions were beneficial, but music with a nursing presence provided a more positive experience.
Janelli et al [61], 2004	Preferred music out of restraints No music out of restraints (control) Preferred music in restraints	11	Observation of positive and negative behaviors during the music intervention phase	Listening to preferred music showed no significant decrease in negative behaviors or increase in positive behaviors for patients in restraints. Patients out of restraints with preferred music had higher positive behavior scores and lower negative behavior scores, suggesting some potential benefit.
Kang et al [62], 2008	Blocking noise with earplugs (silence group) Patient- selected music (music group) Exposure to ambient noise in the operating room (noise group)	O	Bispectral index was measured 7 times during surgery, along with ambient noise levels	Blocking noise with earplugs reduced bispectral index scores compared to exposure to ambient noise during specific surgical procedures, creating high noise levels. Music was not significantly different from noise exposure. Blocking noise may be more effective than music for reducing sedation levels during surgery with high background noise.
Tsivian et al [63], 2012	Music with headphones Noise-cancelling headphones only Control group with no intervention	O	Pain and anxiety scores (questionnaires) and physiological parameters (blood pressure) before and after the procedure	Pain scores were the lowest in the music group. No significant changes in anxiety scores were observed. Patients in the music group showed a smaller increase in DBP after the procedure compared to the other groups, suggesting a reduced physiological stress response.
Gingras et al [64], 2014	Repetitive drumming and shamanic instructions Repetitive drumming and relaxation instructions Instrumental meditation music and shamanic instructions Instrumental meditation music and relaxation instructions	Mix	Salivary cortisol concentration was measured before and after intervention, a self-reported mood questionnaire was administered before and after intervention, and a postexperiment questionnaire on subjective experiences was conducted	All groups showed a decrease in cortisol regardless of music or instructions. Repetitive drumming with shamanic instructions led to specific subjective experiences (heaviness, decreased HR, and dreamlike experiences) compared to drumming with relaxation instructions. Cortisol response may not be sensitive to the specific intervention (shamanic vs relaxation instructions).

^a^HRV: heart rate variability.

^b^MWSA: Mayer Wave Related Sinus Arrhythmia.

^c^RSA: respiratory sinus arrhythmia.

^d^HR: heart rate.

^e^SBP: systolic blood pressure.

^f^STAI-S: State-Trait Anxiety Inventory.

^g^DBP: diastolic blood pressure.

^h^ASMR: autonomous sensory meridian response.

^i^EEG: electroencephalography.

^j^S-IgA: salivary secretory IgA.

^k^NK: natural killer.

^l^MBM: meditative binaural music.

^m^VM: varying music.

^n^SM: stable music.

^o^ECG: electrocardiography.

^p^PMR: progressive muscle relaxation.

^q^COPD: chronic obstructive pulmonary disease.

^r^BVP-A: blood volume pulse amplitude.

### Sound Per Se

#### Overview

The *sound per se* category encompasses studies focused on the direct impact of various sound types on psychological and physiological outcomes, independent of personal or environmental factors. This category is divided into subcategories based on music styles, music sentiment, and the physical aspects of music, examining how specific genres, emotional tones, and sound structures influence stress-related markers. Nonmusical sounds were also considered.

#### Music Styles

In the *sound per se—music styles* category, studies have consistently shown that different music types can influence relaxation and stress reduction. Umemura and Honda [31] found that classical music reduced HRV, indicating relaxation, while rock and noise had the opposite effect, increasing stress markers. Chafin et al [32] observed that classical music significantly lowered systolic blood pressure following a stress task, while other music styles did not. Labbé et al [33] demonstrated that listening to classical or self-selected relaxing music effectively reduced anxiety and physiological arousal compared to heavy metal or silence. Uğraş et al [34] reported that all music styles reduced preoperative anxiety, with classical Turkish music being the most beneficial. Similarly, Gulnahar and Kupeli [35] showed that Turkish and classical music reduced anxiety during dental procedures more effectively than other styles. Finally, Paszkiel et al [36] found that relaxing music and autonomous sensory meridian response decreased stress levels more quickly than silence, whereas rap music increased stress. These findings highlight the calming effects of classical and relaxing music styles across different contexts.

#### Music Sentiment

In the *sound per se—music sentiment* category, research has focused on the impact of different music sentiments on stress recovery. Hirokawa and Ohira [37] suggested that high-uplifting music might enhance mood by increasing norepinephrine levels, while low-uplifting music could potentially boost feelings of well-being. Sokhadze [38] found that both pleasant and sad music improved physiological recovery from stress more effectively than white noise, indicating that positive emotions generated by music might aid in stress recovery. Suda et al [39] demonstrated that major mode music led to lower salivary cortisol levels compared to minor mode music, implying that music in a major mode may induce emotional responses such as happiness, which are closely linked to stress reduction. These findings indicate the potential of music sentiment for stress recovery and mood regulation.

#### Physical Characteristics of Music

In the *sound per se—physical characteristics* category, studies investigated how specific musical features or elements affect stress and anxiety outcomes. Wiwatwongwana et al [40] found that binaural beats reduced anxiety and systolic blood pressure more effectively than plain music during cataract surgery. Opartpunyasarn et al [41] showed that binaural beat music significantly reduced anxiety and blood pressure in patients undergoing bronchoscopy. Lee-Harris et al [42] reported that meditative binaural music had comparable effects to calm classical music, with age influencing preferences. Gantt et al [43] found that binaural beat technology reduced stress by altering HRV in military personnel. Calamassi et al [44] demonstrated that listening to 432 Hz music lowered anxiety and improved respiratory rate more effectively than 440 Hz music. Finally, Sharma et al [45] reported that music with tempo or octave changes reduced anxiety and decreased low-frequency brainwaves, reduced default mode network activity, and more balanced brain activity compared to stable music without variations.

#### Music Versus Human Voice

In the *music versus human voice* category, studies evaluated the relative effectiveness of music and voice-based interventions on anxiety and physiological responses. Singh et al [46] found that both music and progressive muscle relaxation reduced anxiety and physiological markers in patients with chronic obstructive pulmonary disease, with music showing greater effectiveness. Tang et al [47] reported significant reductions in blood pressure after both audio relaxation and music interventions, with audio relaxation showing a greater impact. Lin et al [48] demonstrated that music therapy was more effective than verbal relaxation or usual care in reducing anxiety and physiological responses in patients with cancer undergoing chemotherapy. Warth et al [50] observed that live music therapy led to greater improvements in vagally mediated HRV compared to a prerecorded mindfulness exercise, highlighting its potential for stress management. Finally, Lee et al [49] found that both music and progressive muscle relaxation improved anxiety and relaxation in patients with cancer, with electroencephalography (EEG) data suggesting different neural mechanisms for each intervention.

### Personal Factors

The *personal factors* category examines how individual characteristics, preferences, and personal involvement influence the effectiveness of sound interventions. Leardi et al [53] found that patients who selected their own music during surgery had lower cortisol levels postoperatively and higher immune function compared to those exposed to standard operating room sounds or new age music. Miller et al [54] showed that joyful music improved endothelial function (flow-mediated dilatation), while anxiety-inducing music decreased flow-mediated dilatation, with the effects comparable to those achieved through exercise or medication. Imbriglio et al [55] reported that guided music listening with relaxing or favorite music reduced muscle effort during awake bruxism episodes in patients with temporomandibular disorders, whereas stressful music increased muscle tension increase. This category highlights the importance of tailored interventions, showing that factors such as personal music selection, emotional connection, and the listener’s psychological state can modulate physiological and emotional outcomes. Studies within this category emphasize that the success of sound-based interventions often depends on aligning the experience with the individual’s preferences and emotional needs.

### Environmental Factors

The *environmental factors* category focuses on the influence of delivery methods and contextual conditions on the effectiveness of sound interventions. This category explores how the medium of delivery (eg, live vs recorded music and broadcast vs headphone playback) and environmental settings (eg, presence of supportive personnel) shape physiological and emotional outcomes. Gelatti et al [56] found that live harp music was more effective than recorded harp music in reducing heart rate and blood pressure during surgery. Bro et al [57] reported a borderline reduction in anxiety with live music during chemotherapy, while prerecorded music showed no significant effect. Lee et al [58] and Kumari et al [59] found that both headphones and broadcast music reduced preoperative anxiety, with no significant difference between the 2 methods, suggesting that broadcast music can be an alternative when headphones are unavailable. Lai et al [60] demonstrated that music interventions with a nurse present improved anxiety, sleep quality, and depression more effectively than recorded music alone. Janelli et al [61] observed no significant changes in the behavior of patients who are restrained and listened to preferred music but noted that patients who were not restrained and listened to music had higher rates of positive behavior. The findings underscore that not only the sound itself but also how and where it is delivered plays a critical role in maximizing the benefits of sound-based interventions.

### Mixed Samples

In the *mixed samples* category, we included only 3% (1/34) of the articles. This intervention is considered mixed because it combines both musical (drumming and music) and voice (shamanic and relaxation guidance) elements represented simultaneously. Gingras et al [64] compared the effects of repetitive drumming with shamanic instructions, relaxation instructions, and instrumental meditation music on cortisol levels and subjective experiences. The study found that all groups showed a decrease in cortisol regardless of the intervention, but repetitive drumming with shamanic instructions led to unique subjective experiences, such as feelings of heaviness and dreamlike states, compared to drumming with relaxation instructions.

### Absence of Sound

In the *absence of sound* category, studies examined the effects of blocking noise compared to music interventions. Kang et al [62] found that blocking noise with earplugs reduced bispectral index scores more effectively than exposure to ambient noise during surgery, while music had no significant difference from noise blocking. This suggests that noise reduction may better aid sedation under noisy conditions. Tsivian et al [63] reported that music with headphones reduced pain perception during prostate biopsy compared to noise-canceling headphones or no intervention. Although anxiety scores remained unchanged, patients in the music group showed smaller increases in diastolic blood pressure, indicating a reduced physiological stress response.

### Negative Effects

In the *negative* category, studies reported increases in stress markers resulting from specific sound interventions. Hirokawa and Ohira [37] found that listening to both high- and low-uplifting music decreased salivary secretory IgA levels, indicating reduced immune function, while silence led to a slight increase in salivary secretory IgA. Imbriglio et al [55] observed that stressful music increased muscle effort during awake bruxism episodes, highlighting the potential for certain music to exacerbate physical stress responses. Paszkiel et al [36] indicated that rap-style music increased stress markers compared to silence. These findings suggest that some sound interventions may have unintended adverse effects on physiological conditions.

## Discussion

### Principal Findings

This scoping review synthesizes current research on the effects of sound interventions on stress reduction in adults, highlighting key factors that contribute to their therapeutic potential. The analysis revealed that music, particularly self-selected relaxing music, consistently demonstrated a capacity to reduce both physiological and psychological markers of stress. Nonmusical sounds, including nature sounds and prosodic features of human speech, showed promise but were less commonly studied. As for body responses, studies frequently reported positive effects on HRV, blood pressure, cortisol levels, and self-reported anxiety. Furthermore, limited research using brain imaging demonstrated that sound interventions could modulate neural activity associated with emotional regulation, suggesting a neurobiological mechanism underlying the stress-relieving effects. Overall, these findings highlight sound therapy’s potential in stress management and underscore the need for further exploration of diverse sound types and individualized approaches.

### Therapeutic Factors of Sounds

The results of this review indicate that sound therapy operates through multiple mechanisms. First, it engages emotional systems to reduce stress. Sentiment, or the emotional tone of the sound, plays a significant role: calming and positive emotional tones, such as those found in soothing music, are frequently associated with reductions in anxiety and stress markers. Tempo-rhythm also emerged as a key factor, with studies showing that slower, steady rhythms often promote relaxation by modulating heart rate and respiratory patterns, while faster tempos can increase arousal. The influence of binaural beats and tempo variations further underscores the importance of this factor in modulating stress responses. These findings align with the 4-level model of sound therapy proposed by Clements-Cortes and Bartel [18], which suggests that sound can impact stress both through learned associations and inherent biological mechanisms.

One key takeaway from this review is the nuanced role of personal factors in determining the effectiveness of sound interventions. Studies where participants were allowed to choose music showed greater stress reduction than those with preselected stimuli. This underscores the importance of individual preferences, emotional involvement, and past experiences in shaping responses to sound therapy. Personalization may thus be crucial in optimizing the therapeutic potential of sound interventions.

While personal preference is often acknowledged as a key factor in stress reduction, deeper cultural influences on sound perception and stress response remain insufficiently studied. As we pointed out earlier at the protocol, some studies focus on country-specific interventions, such as traditional music or poetry, implicitly recognizing cultural context. However, only a few explicitly examine whether the therapeutic effects arise from the sound itself or cultural familiarity and learning [27]. Moreover, the concept of “relaxing” or “soothing” environments is highly context-dependent and shaped by previous exposure, traditional use, and sociocultural meaning. Few studies have examined cross-cultural differences in sound interventions, underscoring the need for research on how various musical, poetic, and speech performance traditions may influence responses across populations.

Environmental factors, such as the method of sound delivery, also play a significant role. Live music therapy, for example, appears to have stronger effects on stress markers than prerecorded music, likely due to the interactive and dynamic nature of live performances. Similarly, interventions that involve a supportive human presence, such as a nurse during a music session, further enhance the therapeutic effect.

We remain cautious about identifying the most effective therapeutic factors in sound therapy due to variability in how studies approach comparisons. Some studies focus on fine-grained distinctions in basic characteristics, such as rhythm or frequency spectrum, while others compare broader intervention clusters, such as music versus guided meditation. This difference in levels of generalization complicates direct comparisons. The reviewed studies provided limited evidence on how nonmusical sounds impact physiological stress. Key therapeutic factors such as tempo-rhythm, sentiment, or frequency, were primarily studied within the framework of music therapy. While it is possible to apply similar analytical frameworks to other sounds, these factors may not fully capture the acoustic characteristics that underlie the psychophysical effects of nonmusical sounds. For example, while rhythm in music typically follows a structured, repetitive pattern, rhythm in spoken voice can be more dynamic and context sensitive. Further research is needed to determine whether these factors contribute to therapeutic effects in both musical and nonmusical sounds, as well as to identify acoustic features specific to nonmusical sounds.

### Body Responses to Sounds

The review highlights several physiological responses associated with sound interventions in stressful conditions, measured using various biomedical technologies. HRV and cortisol levels were among the most frequently assessed markers, with multiple studies indicating that calming interventions, such as soothing music or guided relaxation, can balance autonomic nervous system responses, leading to higher HRV and lower cortisol levels. Other commonly measured physiological indicators included blood pressure, electrodermal activity, and respiratory rate, which collectively reflect physiological responses to stress. These metrics were typically recorded using devices such as electrocardiograms for HRV, blood pressure monitors, and salivary samples for cortisol.

In addition, some studies used advanced neuroimaging techniques, such as EEG, to observe brain activity changes in response to sound interventions. This review included limited studies using brain imaging, generally focused on brain activity associated with emotional regulation. For instance, some EEG data showed increased posterior theta and decreased midfrontal beta-2 activity, indicating stress relief. Another study suggested an increase in alpha wave activity, also associated with relaxation, following music and guided relaxation interventions. These neurophysiological findings support the notion that sound therapy can modulate brain activity linked to stress and emotional processing, providing a neurobiological basis for its therapeutic effects.

### Identified Research Gaps

Despite these promising findings, several gaps in the literature were identified. There is a clear need for more research on nonmusical sound interventions, particularly human voice–based therapies and natural soundscapes. Studies comparing different types of sound interventions, beyond the binary classification of music versus silence, are limited. In addition, more research is required to understand the negative or side effects of sound interventions and the optimal conditions for their application.

The fragmentation of the research field and reliance on fragmented concepts are also notable. For instance, several studies emphasize the benefits of classical music over other styles, although it remains unclear what specific factors, aside from the researchers’ personal preferences and cultural backgrounds, contribute to the perceived effectiveness of one style over another. Considering the limited investigation into personal factors, future studies could be designed to compare the effects of the same music on both native listeners and foreigners unfamiliar with one or another musical tradition, rather than focusing on comparisons between different regional styles.

Moreover, incorporating an artistic perspective can enhance our understanding of how esthetic and emotional engagement could influence stress relief. Insights from experts in arts and traditional culture may help explain why some sound interventions are more effective than others. Interdisciplinary collaboration between neuroscientists, psychologists, and art experts is essential for bridging scientific and artistic approaches. Future research should integrate physiological stress markers with qualitative methods, fostering a more holistic view of how personal and cultural background could shape the perceptual impact of sound.

In general, individual factors, such as personal preferences, cultural background, and previous exposure to sound therapy, have not been thoroughly examined. Future studies should consider these factors to develop more personalized sound interventions tailored to individual needs.

Another gap is the relative lack of brain imaging studies. There is still limited knowledge about the neural mechanisms underlying sound therapy’s effects. Expanding neuroimaging research in this field could offer deeper insights into how different types of sounds impact neuronal activity related to emotional regulation and stress processing, particularly in real-time applications of sound therapy in stress management.

### Strengths and Limitations

One of the key strengths of this scoping review is its comprehensive approach to synthesizing evidence across a variety of sound interventions and stress markers. By including diverse studies, such as RCTs, clinical trials, and laboratory experiments, the review offers a broad perspective on how different types of sound, from music to natural sounds and human voices, can influence stress response in adults. This wide scope provides a valuable foundation for understanding the generalizability of sound interventions in stress management. The review also emphasizes the importance of personal, environmental, and delivery factors, shedding light on the nuances of how sound interacts with different listeners and environments.

Another strength lies in the focus on psychophysiological outcomes. By including studies that measure stress through biomarkers such as HRV, cortisol levels, and blood pressure, alongside self-reported emotional states, the review provides a holistic view of how sound interventions impact both body and mind. This dual focus strengthens the findings by bridging subjective experiences with objective physiological evidence.

However, this review also has some limitations. A key challenge is the considerable variation in study designs, including differences in intervention duration, participant demographics, and measured outcomes. This heterogeneity limits the ability to draw broad conclusions about the effectiveness of sound interventions for stress recovery. Future research should prioritize standardizing intervention protocols, including key design elements such as intervention duration, participant characteristics, and outcome measures. These steps would improve comparability across studies and support future meta-analysis in this field.

Another limitation is our focus on the researchers’ interpretations without direct access to the actual sound samples. As evidenced by data comparison, sound can produce both positive and negative effects, depending on its characteristics. Numerous factors may influence these effects simultaneously. For instance, comparing a heavy metal composition with a Mozart lullaby, it is reasonable to expect differences in style, emotional tone, and frequency spectrum simultaneously. If these compositions were placed in their typical performance contexts, such as a concert hall for the classical music versus a stadium with amplified sound and a large, excited audience for the metal piece, the listening environment would likely impact the physiological response of the listener significantly. Therefore, our framework primarily addresses how current papers conceptualize and investigate sound.

Moreover, the term “classical music” is widely used in the reviewed studies, yet it represents a broad spectrum of musical styles that may evoke diverse responses. While some studies reference well-defined classical works (eg, Mozart or Indian classical music), others use the term more generically, leading to potential inconsistencies in interpretation. As we said before, a major limitation in existing research is the lack of access to original sound samples, making it difficult to assess key acoustic factors such as tempo, instrumentation, or emotional tone. Without standardized reporting, cross-study comparisons remain challenging. A potential direction for future research would be to obtain the sound pieces used in original studies and systematically analyze them.

Finally, the review does not provide insights into the long-term effects of sound interventions, as most studies focus on short-term stress relief. The lack of longitudinal data makes it difficult to assess the sustainability of these interventions over time and their potential cumulative benefits for stress management.

### Conclusions

Our scoping review highlights the therapeutic potential of sound interventions in reducing stress, although their effectiveness is influenced by a combination of personal, environmental, and sound-related factors. Future studies should expand the range of sound types studied and explore personalized approaches. Sound-based interventions hold great promise for noninvasive and affordable stress management. Continued exploration of their mechanisms, long-term effects, and optimal applications will be key for unlocking their full potential in both clinical and nonclinical settings.

## Appendix

Multimedia Appendix 1 PRISMA-ScR checklist.

## Abbreviations

EEG: electroencephalography
HRV: heart rate variability
PRISMA-ScR: Preferred Reporting Items for Systematic Reviews and Meta-Analyses Extension for Scoping Reviews
RCT: randomized controlled trial

## Data Availability

Data sharing is not applicable to this paper as no datasets were generated or analyzed during this study.

## References

[1] Chee ZJ, Chang CY, Cheong JY, Malek FH, Hussain S, de Vries M, Bellato A (2024). The effects of music and auditory stimulation on autonomic arousal, cognition and attention: a systematic review. Int J Psychophysiol.

[2] Taheri L, Jahromi MK, Abbasi M, Hojat M (2017). Effect of recorded male lullaby on physiologic response of neonates in NICU. Appl Nurs Res.

[3] Ma W, Thompson WF (2015). Human emotions track changes in the acoustic environment. Proc Natl Acad Sci U S A.

[4] de Witte M, Pinho AD, Stams G, Moonen X, Bos AE, van Hooren S (2022). Music therapy for stress reduction: a systematic review and meta-analysis. Health Psychol Rev.

[5] Mallik A, Russo FA (2022). The effects of music and auditory beat stimulation on anxiety: a randomized clinical trial. PLoS One.

[6] Schneider N, Schedlowski M, Schürmeyer TH, Becker H (2001). Stress reduction through music in patients undergoing cerebral angiography. Neuroradiology.

[7] Nilsson U, Unosson M, Rawal N (2005). Stress reduction and analgesia in patients exposed to calming music postoperatively: a randomized controlled trial. Eur J Anaesthesiol.

[8] Blacking J (1973). How Musical is Man?.

[9] Colverson AJ, Trifilio E, Williamson JB (2022). Music, mind, mood, and mingling in Alzheimer's disease and related dementias: a scoping review. J Alzheimers Dis.

[10] Thaut M (2005). Rhythm, Music, and the Brain: Scientific Foundations and Clinical Applications.

[11] Tutaj L, Hoare DJ, Sereda M (2018). Combined amplification and sound generation for tinnitus: a scoping review. Ear Hear.

[12] Thaut MH (2015). Music as therapy in early history. Prog Brain Res.

[13] Mohammadian Y, Shahidi S, Mahaki B, Mohammadi AZ, Baghban AA, Zayeri F (2011). Evaluating the use of poetry to reduce signs of depression, anxiety and stress in Iranian female students. Arts Psychother.

[14] Jabarouti R, Shariat A, Shariat A (2014). Effect of Persian classic poetry on the level of stress hormone in retired academicians. J Poet Ther.

[15] Shafi N (2010). Poetry therapy and schizophrenia: clinical and neurological perspectives. J Poet Ther.

[16] Ackerman SJ, Hilsenroth MJ (2003). A review of therapist characteristics and techniques positively impacting the therapeutic alliance. Clin Psychol Rev.

[17] Soma CS, Knox D, Greer T, Gunnerson K, Young A, Narayanan S (2023). It's not what you said, it's how you said it: an analysis of therapist vocal features during psychotherapy. Couns Psychother Res.

[18] Clements-Cortes A, Bartel L (2018). Are we doing more than we know? Possible mechanisms of response to music therapy. Front Med (Lausanne).

[19] Song I, Baek K, Kim C, Song C (2023). Effects of nature sounds on the attention and physiological and psychological relaxation. Urban For Urban Green.

[20] Zhu R, Yuan L, Pan Y, Wang Y, Xiu D, Liu W (2024). Effects of natural sound exposure on health recovery: a systematic review and meta-analysis. Sci Total Environ.

[21] Bliss-Moreau E, Barrett LF, Owren MJ (2010). I like the sound of your voice: affective learning about vocal signals. J Exp Soc Psychol.

[22] de Witte M, Orkibi H, Zarate R, Karkou V, Sajnani N, Malhotra B, Ho RT, Kaimal G, Baker FA, Koch SC (2021). From therapeutic factors to mechanisms of change in the creative arts therapies: a scoping review. Front Psychol.

[23] Sexton TL (2007). The therapist as a moderator and mediator in successful therapeutic change. J Fam Ther.

[24] Shen X, Masek L (2023). The playful mediator, moderator, or outcome? An integrative review of the roles of play and playfulness in adult-centered psychological interventions for mental health. J Posit Psychol.

[25] Kazdin AE (2009). Understanding how and why psychotherapy leads to change. Psychother Res.

[26] Robb SL, Stegenga K, Perkins SM, Stump TE, Moody KM, Henley AK, MacLean J, Jacob SA, Delgado D, Haut PR (2023). Mediators and moderators of active music engagement to reduce traumatic stress symptoms and improve well-being in parents of young children with cancer. Integr Cancer Ther.

[27] Saskovets M, Liang Z, Piumarta I, Saponkova I (2024). Effects of sound interventions on the mental stress response in adults: protocol for a scoping review. JMIR Res Protoc.

[28] Tricco AC, Lillie E, Zarin W, O'Brien KK, Colquhoun H, Levac D, Moher D, Peters MD, Horsley T, Weeks L, Hempel S, Akl EA, Chang C, McGowan J, Stewart L, Hartling L, Aldcroft A, Wilson MG, Garritty C, Lewin S, Godfrey CM, Macdonald MT, Langlois EV, Soares-Weiser K, Moriarty J, Clifford T, Tunçalp Ö, Straus SE (2018). PRISMA extension for scoping reviews (PRISMA-ScR): checklist and explanation. Ann Intern Med.

[29] Pollock D, Peters MD, Khalil H, McInerney P, Alexander L, Tricco AC, Evans C, de Moraes ÉB, Godfrey CM, Pieper D, Saran A, Stern C, Munn Z (2023). Recommendations for the extraction, analysis, and presentation of results in scoping reviews. JBI Evid Synth.

[30] Braun V, Clarke V (2006). Using thematic analysis in psychology. Qual Res Psychol.

[31] Umemura M, Honda K (1998). Influence of music on heart rate variability and comfort--a consideration through comparison of music and noise. J Hum Ergol (Tokyo).

[32] Chafin S, Roy M, Gerin W, Christenfeld N (2004). Music can facilitate blood pressure recovery from stress. Br J Health Psychol.

[33] Labbé E, Schmidt N, Babin J, Pharr M (2007). Coping with stress: the effectiveness of different types of music. Appl Psychophysiol Biofeedback.

[34] Uğraş GA, Yıldırım G, Yüksel S, Öztürkçü Y, Kuzdere M, Öztekin SD (2018). The effect of different types of music on patients' preoperative anxiety: a randomized controlled trial. Complement Ther Clin Pract.

[35] Gulnahar Y, Kupeli I (2020). Effect of different kinds of music on anxiety during implant surgery in Turkey: randomized controlled study. Int J Oral Maxillofac Implants.

[36] Paszkiel S, Dobrakowski P, Łysiak A (2020). The impact of different sounds on stress level in the context of EEG, cardiac measures and subjective stress level: a pilot study. Brain Sci.

[37] Hirokawa E, Ohira H (2003). The effects of music listening after a stressful task on immune functions, neuroendocrine responses, and emotional states in college students. J Music Ther.

[38] Sokhadze EM (2007). Effects of music on the recovery of autonomic and electrocortical activity after stress induced by aversive visual stimuli. Appl Psychophysiol Biofeedback.

[39] Suda M, Morimoto K, Obata A, Koizumi H, Maki A (2008). Emotional responses to music: towards scientific perspectives on music therapy. Neuroreport.

[40] Wiwatwongwana D, Vichitvejpaisal P, Thaikruea L, Klaphajone J, Tantong A, Wiwatwongwana A (2016). The effect of music with and without binaural beat audio on operative anxiety in patients undergoing cataract surgery: a randomized controlled trial. Eye (Lond).

[41] Opartpunyasarn P, Vichitvejpaisal P, Oer-Areemitr N (2022). The effect of binaural beat audio on anxiety in patients undergoing fiberoptic bronchoscopy: a prospective randomized controlled trial. Medicine (Baltimore).

[42] Lee-Harris G, Timmers R, Humberstone N, Blackburn D (2018). Music for relaxation: a comparison across two age groups. J Music Ther.

[43] Gantt MA, Dadds S, Burns DS, Glaser D, Moore AD (2017). The effect of binaural beat technology on the cardiovascular stress response in military service members with postdeployment stress. J Nurs Scholarsh.

[44] Calamassi D, Li Vigni ML, Fumagalli C, Gheri F, Pomponi GP, Bambi S (2022). The listening to music tuned to 440 Hz versus 432 Hz to reduce anxiety and stress in emergency nurses during the COVID-19 pandemic: a double-blind, randomized controlled pilot study. Acta Biomed.

[45] Sharma S, Sasidharan A, Marigowda V, Vijay M, Sharma S, Mukundan CS, Pandit L, Masthi NR (2021). Indian classical music with incremental variation in tempo and octave promotes better anxiety reduction and controlled mind wandering-a randomised controlled EEG study. Explore (NY).

[46] Singh VP, Rao V, V P, R C S, K KP (2009). Comparison of the effectiveness of music and progressive muscle relaxation for anxiety in COPD--a randomized controlled pilot study. Chron Respir Dis.

[47] Tang HY, Harms V, Speck SM, Vezeau T, Jesurum JT (2009). Effects of audio relaxation programs for blood pressure reduction in older adults. Eur J Cardiovasc Nurs.

[48] Lin MF, Hsieh YJ, Hsu YY, Fetzer S, Hsu MC (2011). A randomised controlled trial of the effect of music therapy and verbal relaxation on chemotherapy-induced anxiety. J Clin Nurs.

[49] Lee EJ, Bhattacharya J, Sohn C, Verres R (2012). Monochord sounds and progressive muscle relaxation reduce anxiety and improve relaxation during chemotherapy: a pilot EEG study. Complement Ther Med.

[50] Warth M, Kessler J, Hillecke TK, Bardenheuer HJ (2016). Trajectories of terminally ill patients' cardiovascular response to receptive music therapy in palliative care. J Pain Symptom Manage.

[51] Koehler F, Kessler J, Stoffel M, Weber M, Bardenheuer HJ, Ditzen B, Warth M (2022). Psychoneuroendocrinological effects of music therapy versus mindfulness in palliative care: results from the 'Song of Life' randomized controlled trial. Support Care Cancer.

[52] Radstaak M, Geurts SA, Brosschot JF, Kompier MA (2014). Music and psychophysiological recovery from stress. Psychosom Med.

[53] Leardi S, Pietroletti R, Angeloni G, Necozione S, Ranalletta G, Del Gusto B (2007). Randomized clinical trial examining the effect of music therapy in stress response to day surgery. Br J Surg.

[54] Miller M, Mangano CC, Beach V, Kop WJ, Vogel RA (2010). Divergent effects of joyful and anxiety-provoking music on endothelial vasoreactivity. Psychosom Med.

[55] Imbriglio TV, Moayedi M, Freeman BV, Tenenbaum HC, Thaut M, Cioffi I (2020). Music modulates awake bruxism in chronic painful temporomandibular disorders. Headache.

[56] Gelatti F, Viganò C, Borsani S, Conistabile L, Bonetti L (2020). Efficacy of live versus recorded harp music in reducing preoperative stress and fear related to minor surgery: a pilot study. Altern Ther Health Med.

[57] Bro ML, Johansen C, Vuust P, Enggaard L, Himmelstrup B, Mourits-Andersen T, Brown P, d'Amore F, Andersen EA, Abildgaard N, Gram J (2019). Effects of live music during chemotherapy in lymphoma patients: a randomized, controlled, multi-center trial. Support Care Cancer.

[58] Lee KC, Chao YH, Yiin JJ, Chiang PY, Chao YF (2011). Effectiveness of different music-playing devices for reducing preoperative anxiety: a clinical control study. Int J Nurs Stud.

[59] Kumari S, Singh RK, Harshwardhan (2023). Effectiveness of different music-playing devices for reducing preoperative anxiety. Int J Curr Pharm Rev Res.

[60] Lai HL, Li YM, Lee LH (2012). Effects of music intervention with nursing presence and recorded music on psycho-physiological indices of cancer patient caregivers. J Clin Nurs.

[61] Janelli LM, Kanski GW, Wu YW (2004). The influence of individualized music on patients in physical restraints: a pilot study. J N Y State Nurses Assoc.

[62] Kang JG, Lee JJ, Kim DM, Kim JA, Kim CS, Hahm TS, Lee BD (2008). Blocking noise but not music lowers bispectral index scores during sedation in noisy operating rooms. J Clin Anesth.

[63] Tsivian M, Qi P, Kimura M, Chen VH, Chen SH, Gan TJ, Polascik TJ (2012). The effect of noise-cancelling headphones or music on pain perception and anxiety in men undergoing transrectal prostate biopsy. Urology.

[64] Gingras B, Pohler G, Fitch WT (2014). Exploring shamanic journeying: repetitive drumming with shamanic instructions induces specific subjective experiences but no larger cortisol decrease than instrumental meditation music. PLoS One.

